# District Nursing

**Published:** 1920-07-03

**Authors:** 


### District Nursing.

**The Chairman (Sir Wm. Cameron Gull,** Bart., O.B.E.) announced that Miss Peterkin was unable to be present, and that Miss Clarke had written urging that district nurses should have an adequate uniform, with provision for bad weather, an increase of salary, and that there should be more confidence placed in the nurse, with a lessening of the critical hindrance of inspectors.

The District Nurse's Unique Position.

**Miss E. Hancox** (Lady Superintendent, Q.V.J.N.I., Sheffield) said there had been a very rapid development of district nursing recently, when a different type of woman had been induced to take up the work. The history of this sphere of nursing work was closely interwoven with that of civic hygiene, and now the district nurse was nothing if not up to date; she must be well up in all the branches of her profession, and be *au fait* with all the Acts and amendments to Acts bearing upon public health. The district nurse must be broad-minded and sympathetic, and a woman of sound common sense; it was not everyone who could be a district nurse and who could adapt herself to circumstances by self-abnegation and the exercise of tact. She must not only herself be courageous, but able to inspire courage in others. The district nurse got to know the people more than did any other kind of nurse, and she took an interest in their home affairs, because she was looked up to as their real friend. Never was there such a demand for the district nurse as now; her scope was enormous, her influence unique. Sir George Newman had well said that we must make available for all that better nursing and closer home attention which had hitherto been the lot of only the favoured few, and that sentence applied specially to the case of the district nurse. Domiciliary nursing was an essential part of health service. She pleaded for the support of the matrons of hospitals; often the attention of the district nurse in the home made possible a much earlier discharge of patients from hospital.

**Miss G. R. Skinner** (County Superintendent, Berkshire Nursing Association) spoke on the rural aspect of the subject, emphasising the importance of the district nurse being well protected from the bad weather. In rural districts especially the working life of the district nurse was very short, and, in view of the depressing nature of much of her work, she ought to have good housing accommodation and opportunities for recreation. In rural areas especially the district nurse should be able to act in an emergency, for the nearest doctor might be many miles away. She wished to pay a special tribute to the village nurse-midwife, who did much really good work. The superintendent did not accompany the nurse in order to criticise, but to help; she had had more experience, and therefore was able to show how things should be done.

**Miss Tait McKay, R.R.C.** (late Matron, 5th Southern General Hospital, Plymouth), spoke of the need for the district nurse having a large outlook. In the present unrest and desire for reconstruction, the people's guides must have the courage to discard the obsolete and grasp the good in everything. She applied these tenets to combating such social evils as venereal disease, drink, bad housing, and high infant mortality. The crucifying of a woman's self-respect after her frailty had been exploited received her strongest condemnation. She urged that the training of nurses should be remodelled; in three or four years the training institutions ought to be able to turn out nurses able to work in any nursing sphere.

**Miss Gane** (Somerset) contested the view that the district nurse was so isolated as seemed to be thought. She agreed that the support of hospital matrons should be given to district nurses. Fully trained nurses were naturally preferable, but there were not enough of them, and meantime partially trained women were valuable.

**The Chairman** said the district nurse was doing a tremendous lot of preventive work in the widest sense of the term; she had been the best influence in changing the character of homes and districts. In the country districts—of which he had better knowledge than of towns—it was a nuisance to have various officials entering people's homes; there ought to be more co-ordination. In the counties the authorities were in a parlous state in regard to staffs to carry out the needed work. He agreed that the salaries paid to nurses were wholly inadequate. He suggested the establishment of emergency organisations in country districts, which would be ready to deal with epidemics or other similar emergencies.

